# Epidemiology of Chemical Poisoning among Adults in Qassim Region: An Eight-Year Study

**DOI:** 10.3390/toxics10110709

**Published:** 2022-11-21

**Authors:** Sulaiman Mohammed Alnasser, Tamader Saeed Kordi, Ali Ahmad Asiri, Dhirendra Kumar Gupta, Abubakr Abdelraouf Alfadl, Abubakar Siddiqui Mustafa Hussain

**Affiliations:** 1 Department of Pharmacology and Toxicology, Unaizah College of Pharmacy, Qassim University, Qassim 51911, Saudi Arabia; 2 Office of Assistant Deputyship for Preventive Health, Ministry of Health, Buraidah 51911, Saudi Arabia; 3 General Dirtectorate of Environmental Health, Ministry of Health, Buraidah 51911, Saudi Arabia; 4 Deanship of Educational Services, Qassim University, Qassim 51911, Saudi Arabia; 5 Department of Pharmacy Practice, Unaizah College of Pharmacy, Qassim University, Qassim 51911, Saudi Arabia

**Keywords:** adults, chemical poisoning, poisoning agent, Saudi Arabia

## Abstract

Chemical poisoning is considered a common medico-social problem that, in addition to causing extensive morbidity and mortality, dominates the valuable health care service resources. Therefore, this study was conducted to explore the extent and frequency of chemical poisoning events among adults in Qassim region as well as the most common poisoning agents involved. A retrospective method of data collection was used employing medical record review for chemical poisoning cases that occurred in Qassim region during the 8-year period from January 2008 to December 2015. Data were collected using a standardized, validated data collection sheet. The study revealed that there is no steady trend (either decreasing or increasing) of the number of poisoning cases through time. There is a statistically significant association between the type of poisoning agent and gender (χ^2^ = 14.3104, *p* < 0.05). Moreover, there is a statistically significant association between the type of poisoning agent and period in years (χ^2^ = 19.7565, *p* < 0.05). It can be concluded that poisoning cases are distributed, to some extent, evenly between males and females, with no pattern through time. Educational programs are needed to raise public awareness about poisoning, especially among women.

## 1. Introduction

Any substance that can harm living beings as a result of absorption into the body, following contact with the skin or after ingestion, is known as a poison. Poisonous materials that cross the placenta can affect the fetus as well. Many substances can act as poisons if a sufficiently large dose is absorbed [1]. These poisons result in chemical actions which are capable of producing injury or dysfunction in the body [2,3]. It is documented that poisoning represents a significant global public health challenge in terms of morbidity and mortality to the extent that it is considered the second largest cause of morbidity worldwide, following road traffic accidents [2]. Therefore, poisoning is considered a common medico-social issue that, in addition to causing extensive morbidity and mortality, dominates the valuable health care service resources [4]. Estimations attribute more than 340,000 unintentional deaths to poisoning which can result in the loss of more than 7.4 million years of healthy life globally (disability adjusted life years, DALYs) [2]. In addition to unintentional incidents, the estimation of intentional poisoning cases gives a number of about 2 million per year, causing an approximate 200,000 associated deaths [5].

However, in spite of all that, poisoning is still considered as a neglected problem [6]. This has resulted in few researches having investigating this problem, leading to inadequate available epidemiological data, especially in developing countries including Saudi Arabia. Consequently, the exact extent and complication of the problem is not known. Therefore, this study was conducted to explore the extent and frequency of chemical poisoning events among adults in Qassim region as well as the most common poisoning agents involved to fill this literature gap, especially in Saudi Arabia, as it is essential for well-planned intervention for mitigating the problem to have a clear picture about the nature and magnitude of the problem.

**Figure 1:**
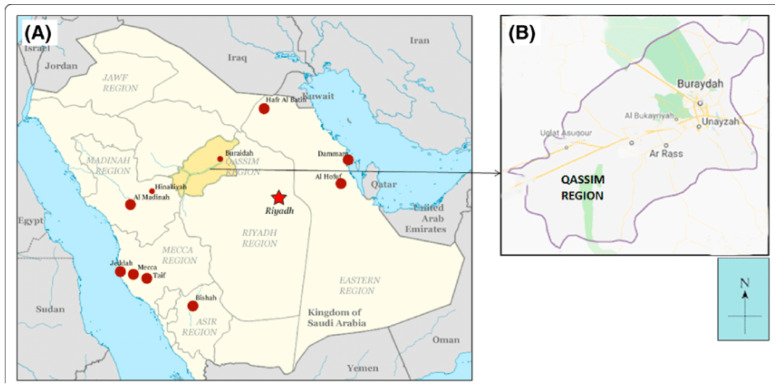
Location of Qassim in Saudi Arabia.

In Saudi Arabia, health services have developed enormously over the last two decades, as evidenced by the availability of health facilities throughout all parts of the entire Kingdom. Health care services were provided mainly by the Ministry of Health (MoH) through a large network of Primary Health Care (PHC) centers and hospitals across the country [7]. In Qassim, in particular, which is located in the central part of Saudi Arabia (Figure 1), there are 19 hospitals under MoH, one hospital under other governmental sector and four private hospitals. The number of MoH PHCs is 156 while there are 119 private polyclinics providing health care services under different specialties in the region [8]. These facilities serve a population of 1.5 million as estimated in 2020.

However, despite the development in healthcare, during last decade Saudi Arabia has witnessed economic and financial structural reforms that have negatively impacted population health. The economic development plans focused on the industrial sector to diversify the production base and to eliminate the effects of dependence on oil as the sole source of national income. These efforts resulted in the development of the manufacturing sector. During the past four decades, the industrial base in Saudi Arabia has expanded considerably, increasing the number of factories from 206 in 1974 to 7630 in 2018. The number of workers increased from about 10,000 in 1974 to more than one million in 2018 [9]. With this continuous growth of the industrial sector throughout this period, chemicals production continues to increase and, with it, the potential for chemical exposure.

## 2. Materials and Methods

This study was a retrospective review of chemical poisoning cases that occurred during the 8-year period from January 2008 to December 2015. Data about poisoning cases were extracted from 381 medical records with no intervention or any direct contact with patients. The cases were reported to the Food and Chemical Safety Program, Environmental and Occupational Health Directorate, General Directorate of Public Health, Ministry of Health (MOH), Saudi Arabia as part of a national program of food, drug and chemical safety. The purpose of the program is to record the adverse effects of food, drug and chemical poisoning cases on humans during production, storage, transport and use. The reporting system is mandatory for each poisoning case by all health care providers. All cases are saved in Microsoft Excel. The study has received ethical approval from Qassim Research Ethics Committee, Saudi Arabia (No. 20,170,415 Dated: 27 April 2017). A data collection sheet constructed by the consultant of the program (the first author) was used to record personal and socio-demographic information of the poison category (detergents, disinfectants, insecticides and pesticides, fuels, others), and date of the incident.

Data wereanalyzed by SPSS software version 20. Descriptive statistics were generated as simple frequency tables and figures. For inferential statistics, chi-square tests were used to compare categorical variables. Statistical significance was set at *p* < 0.05.

## 3. Results

**Figure 2:**
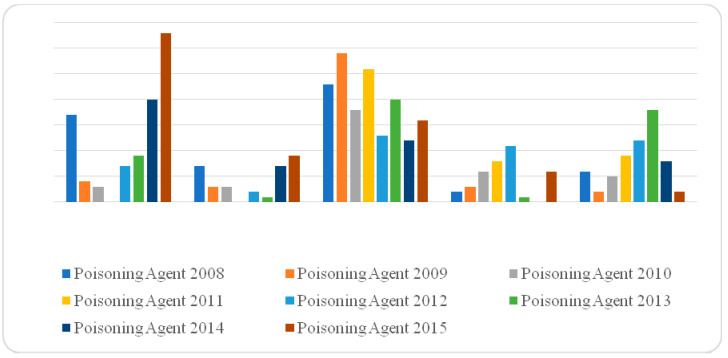
Frequency of poisonings and the type of poison ingested according to period of poisoning.

**Figure 3:**
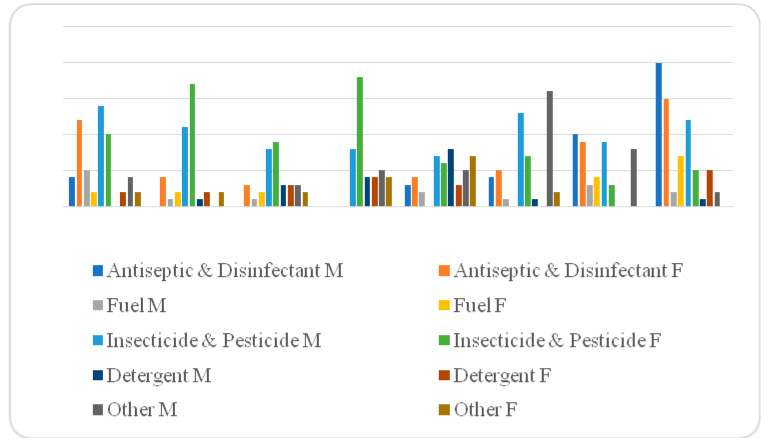
Frequency of poisonings and the type of poison ingested according to gender and period of poisoning.

During the period from January 2008 to December 2015, 381 poisoning cases were reported to the Environmental and Occupational Health Directorate, Riyadh, after being admitted to hospitals as a consequence of a poisoning event. The highest number of poisoning cases was reported during the year 2015. However, no steady trend (either decreasing or increasing) of the number of poisoning cases could be reported overtime. Instead, the trend is zigzagging up and down as can be shown in Figure 2 and Figure 3 below.

The sample is balanced gender wise with 52.2% male and 47.8% female. About three quarters (76.4%) of the poisoning cases occurred among Saudi citizens. Moreover, about three quarters (71.7%) of the cases were accidental, while less than one quarter (22.6%) were intentional. Almost all poisoned victims experienced a complete recovery (97.4%). Characteristics of chemical poisoning were detailed in Table 1 below.

Results revealed statistically significant associations between the type of poisoning agent and gender (χ^2^ = 14.3104, *p* < 0.05). The rate of poisoning by substances labelled “Other” among males (21.61%) was more than double the rate among females (10.44%). Details of the findings of associations between frequency and type of poisoning agent and gender were presented in Table 2 below.

Moreoever, statistically significant associations (χ^2^ = 19.7565, *p* < 0.05) between the type of poisoning agent and period in years were shown. The number of poisoning cases due to antiseptic and disinfectant were the highest in the years 2014–2015 (54), while the highest number of poisoning cases (70 cases) in the years 2008–2010 was due to insecticide agents. Details of the findings of associations between frequency and type of poisoning agent and period were presented in Table 3 below.

## 4. Discussion

Despite the many health statistics collected from the community in Qassim province, scarce information is available about the frequency of poisoning cases and type of poisoning agents involved. Consequently, no formal registry exists to document this important information. This is one of the factors that encouraged Environmental and Occupational Health Directorate, Qassim, to exploit admissions to casualty departments and hospital wards as a tool for collection and documentation of such important missing information.

Results of this study showed that insecticides followed by antiseptic and disinfectants, detergents, and then fuel were the most common agents of chemical poisoning in Qassim region. Similar findings were reported from Nepal [10], Al Majmaah [11], India [12], Zambia [2], and China [13]. This agreement in literature about most common chemical poisoning agents seems logical as exposure with these toxic materials increased due to increased development in the industrial and agricultural field that had led to the wider availability of insecticides [14].

The literature reported a rising trend in the number of incidences of chemical poisoning generally, and pesticides poisoning specifically. Previous studies estimated the number of deaths due to toxicities caused by pesticides and natural toxins to be about 500,000 per year. Up to half of these poisoning events caused by pesticides occurred in the developing countries despite the fact that these countries account for only 15% of the worldwide use of pesticides [15]. This clearly highlights the weak awareness about handling of these poisoning agents among communities of developing countries. Previous studies conducted in Saudi Arabia and elsewhere highlighted the same [16,17].

In contradiction with the results of this study, a researcher from Makkah reported that detergents were the most common poisoning agents [18]. These inconsistencies may be attributed to socio-economic differences. It is possible that pesticides and insecticides are the most common poisoning agents in our study because of the agriculturally-based populations. These results support the previous literature claiming that chemical intoxication demonstrates geographical variations and is also influenced by the socio-economic level of the society [11].

An interesting finding was that more than one-fifth of the poisoning cases was intentional. However, similar rates were reported in other regions of Saudi Arabia and elsewhere [10,12,19,20]. Moreover, a similar rate of death as was reported in this current study was documented in the literature [2,10,18,21].

The study also revealed a statistically significant association between gender and type of poisoning. A slight predominance in the number of poisoning cases was observed among females compared with males. This contradicts findings obtained from an Indian tertiary care hospital [22]. Furthermore, a recent study conducted in Jeddah, Saudi Arabia also contradictsthe findings from this study [20]. However, the current finding of this study had support from several studies conducted in the National Guard Hospital in Jeddah, Al-Qassim, Riyadh, India, and Malaysia reported a higher rate among females [18,23,24]. It was also noticed that females were more prone to being poisoned by antiseptics and disinfectants. On the other hand, males were more vulnerable to “Other” poisonous agents. This could be attributed to the fact that women in Qassim spend most of their time at home with families, making them more prone to indoor chemical poisoning. On the other hand, males of working age tend to spend most of their time in outdoor activities, making them more vulnerable to other types of chemical substances. Findings similar to those obtained from this study were also reported in other local, as well as international studies [2,13,20].

The findings of this study also revealed significant associations between types of poisoning agents involved in poisoning cases and the period of years included in the study. However, this finding not only has no support from the previous literature, but moreover, contradicts some other studies [3]. In addition, as no steady trend can be observed, it is difficult to attribute these fluctuations up and down in the number of cases for each type of poisoning agent to changes in lifestyle or social behaviour of the society through time.

The main limitation of our study is that not all information was reported to the program. We were unable to measure certain key statistics including hospitalization rate and duration, specific poisoning antidotes, specific place where the poisoning occurred, and employment of patients which were not reported to the program.

## 5. Conclusions

In conclusion, most cases of poisoning affected females with no pattern through time. Our results indicate that more educational programs are needed to increase the awareness of the public about chemical poisoning and itseffects. These programs can be delivered through mass media or in shopping malls as most of the customers of these shopping malls are females. Such programs need to focus specifically on recommendations for suitable ways of storing chemical products.

## Figures and Tables

**Table 1:** Characteristics of chemical poisoning cases according to the study variables.

Variables	Cases	
	Number	%
Gender		
Male	199	52.2
Female	182	47.8
Nationality		
Saudi	291	76.4
Non Saudi	90	23.6
Type of Exposure		
Accidental	273	71.7
Intentional	86	22.6
Unknown	21	5.5
Exposure route		
Oral	335	87.9
Dermal	16	4.2
Unknown	30	7.9
Laboratory results		
Positive detection of drug poisoning	45	11.8
Negative detection of drug poisoning	30	7.9
No request	306	80.3
Outcomes		
Recovery	371	97.4
Death	10	2.6

**Table 2:** Relationship between gender and frequency and type of poison ingested.

Variables			Poisoning Agent					Total	χ^2^
			Antiseptic & Disinfectant	Fuel	Insecticide	Cleansing Substance	Other		(*p*)
Gender	Male	Count	40	15	83	18	43	199	14.3104 (<0.05)
		% within Poisoning Agent	20.10	7.54	41.71	9.05	21.61	100.00	
	Female	Count	53	17	74	19	19	182	
		% within Poisoning Agent	29.12	9.34	40.66	10.44	10.44	100.00	
Total		Count	93	32	157	37	62	381	
		% within Poisoning Agent	24.41	8.40	41.21	9.71	16.72	100.00	

**Table 3:** Relationship between time period and frequency and type of poison ingested.

Variables			Poisoning Agent					Total	χ^2^
			Antiseptic & Disinfectant	Fuel	Insecticide	Cleansing Substance	Other		(*p*)
Duration	2008–2010	Count	23	13	70	11	13	130	19.7565 (<0.05)
		% within Poisoning Agent	17.69	10.00	53.85	8.46	10.00	100	
	2011–2013	Count	16	3	59	20	39	137	
		% within Poisoning Agent	11.68	2.19	43.07	14.60	28.47	100	
	2014–2015	Count	54	16	28	6	10	114	
		% within Poisoning Agent	47.37	14.04	24.56	5.26	8.77	100	
Total		Count	93	32	157	37	62	381	
		% within Poisoning Agent	24.41	8.40	41.21	9.71	16.27	100	

## Data Availability

The data that support the findings of this study are availablefrom the corresponding author, S.M.A., upon reasonable request.
